# Definition of Post–COVID-19 Condition Among Published Research Studies

**DOI:** 10.1001/jamanetworkopen.2023.5856

**Published:** 2023-04-05

**Authors:** Ubonphan Chaichana, Kenneth K. C. Man, Anthony Chen, Ian C. K. Wong, Jacob George, Peter Wilson, Li Wei

**Affiliations:** 1 UCL School of Pharmacy, London, United Kingdom; 2 Laboratory of Data Discovery for Health (D24H), Hong Kong Science Park, Hong Kong Special Administrative Region, China; 3 Centre for Medicines Optimisation Research and Education, University College London Hospitals National Health Service (NHS) Foundation Trust, London, United Kingdom; 4 Centre for Safe Medication Practice and Research, Department of Pharmacology and Pharmacy, Li Ka Shing Faculty of Medicine, The University of Hong Kong, Hong Kong Special Administrative Region, China; 5 Ninewells Hospital, University of Dundee Medical School, Dundee, United Kingdom; 6 University College London Hospitals NHS Foundation Trust, London, United Kingdom

## Abstract

This cross-sectional study examines the differing definitions of a post–COVID-19 condition among published studies.

## Introduction

As of February 2023, there have been approximately 759 million confirmed cases of COVID-19 infections globally^1^ and some individuals have experienced persistent symptoms, such as fatigue and shortness of breath, after recovering from the initial illness from COVID-19. The UK National Institute for Health and Care Excellence (NICE),^2^ the World Health Organization (WHO),^3^ and the US Centers for Disease Control and Prevention (CDC)^4^ have published their definitions of post–COVID-19 condition (PCC) between December 2020 and October 2021, with some discrepancies between them. Despite the growing volume of research on lasting symptoms of COVID-19, the definition has not been universally agreed on. This study aimed to describe how post–COVID-19 condition has been defined to date in studies on this topic.

## Methods

We conducted a descriptive study on PCC definition following the STROBE reporting guideline and performed the literature search using the PRISMA checklist in PubMed on October 26, 2022. A total of 7087 studies containing information on PCC were identified from February 1, 2020, to October 26, 2022. Definition of PCC (eAppendix in Supplement 1), study type, country where the study was conducted, and manuscript submission date were extracted from the publications and are presented chronologically (eAppendix in Supplement 1).

Two investigators (U.C. and A.C.) reviewed the studies and screened titles and abstracts independently and cross-checked a 10% sample of the data collected from the studies. When submission dates were not available, the publication dates were used to determine the study time. Exemption from ethical approval was indicated by the University College of London Ethics Committee. SPSS Statistics for Windows, version 28 (IBM Corp) was used for data analysis.

## Results

**Figure 1.**
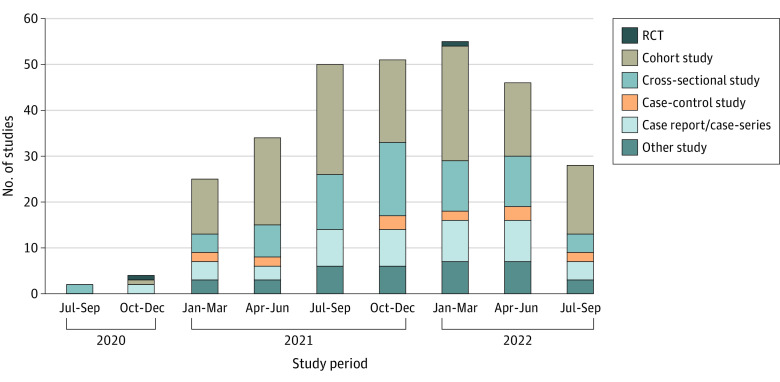
The Proportion of Post–COVID-19 Condition Study Designs Over Time RCT indicates randomized clinical trial.

**Figure 2.**
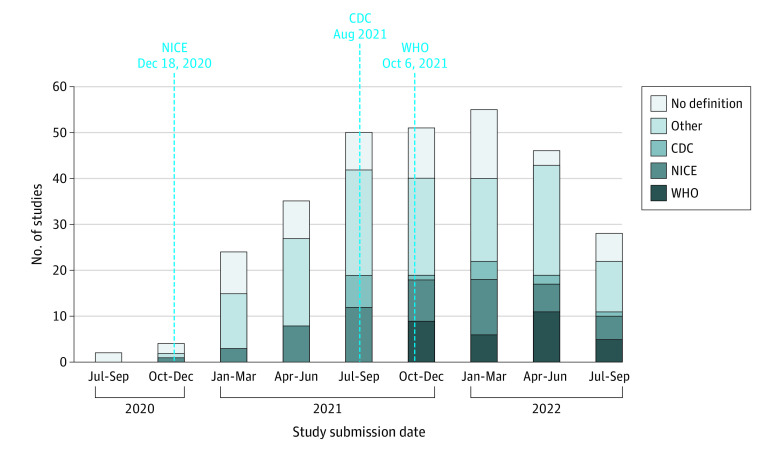
Use of Post–COVID-19 Condition Definition by Organization and Submission Date CDC indicates Centers for Disease Control and Prevention; NICE, UK National Institute for Health and Care Excellence; WHO, World Health Organization.

Among 7087 studies, we excluded 6792 that were not relevant to PCC (eg, SARS-CoV-2 vaccines, commentary, systematic review, and full articles in languages other than English). The remaining 295 studies were included, consisting of 2 randomized clinical trials (0.7%), 134 cohort studies (45.4%), 66 cross-sectional studies (22.4%), 13 case-control studies (4.4%), 45 case reports or case series (15.3%), and 35 studies using other designs (11.9%) (Figure 1). Of these, 167 studies (56.6%) were conducted in European countries. We found that only 102 studies (34.6%) used 1 of the 3 organizational definitions for their studies (NICE: 56, WHO: 31, and CDC: 15). A total of 193 studies (65.4%) did not follow any of the 3 definitions for PCC and 6 studies were submitted for publication before NICE released their PCC definition (ie, before December 18, 2020) (Figure 2).

Of 193 studies that did not follow any of 3 definitions, 129 studies (66.8%) used their own definitions for PCC (eg, presence of chronic symptoms that last >5 months or after 2 weeks of SARS-CoV-2 infection), while 64 studies (33.2%) did not define PCC.

## Discussion

We found substantial heterogeneity in defining PCC in the published studies, with almost two-thirds (65.4%) not complying with the definitions from the NICE, CDC, or WHO. This study highlights major issues in comparing interventions and outcomes between these reported studies in PCC due to differences in definition. The differences also result in considerable variation when translating findings into clinical management and cost-effectiveness assessments of interventions in patients with PCC. The clinical management of PCC must be evidence-based and include a personalized approach. A clearer definition of PCC is timely so that clinical trial evidence can reliably be applied to clinical management and the well-being of patients with PCC can be improved.

Our study has some limitations. We conducted the literature search only in PubMed. Furthermore, the NICE updated their PCC definition in November 2022 after we finished the study screening. However, the updated definition would not affect our study and would only apply to studies conducted after November 2022.

## Notes

Supplement 1. **eAppendix.** Search Strategy

Supplement 2.
Data Sharing Statement
