# Supplementary material for: Definition of Post–COVID-19 Condition Among Published Research Studies
Source: JAMA Netw Open. 2023 Apr 5;6(4):e235856. doi: 10.1001/jamanetworkopen.2023.5856 (PMC10077105; doi:10.1001/jamanetworkopen.2023.5856)
Supplement: Supplement 1. — eAppendix. Search Strategy [file jamanetwopen-e235856-s001.pdf]

Supplementary Online Content

Chaichana U, Man KKC, Chen A, et al. Definition of post–COVID-19 condition among published research studies. *JAMA Netw Open*. 2023;6(4):e235856. doi:10.1001/jamanetworkopen.2023.5856

eAppendix. Search Strategy

This supplementary material has been provided by the authors to give readers additional information about their work.

© 2023 Chaichana U et al. *JAMA Network Open.*

eAppendix. Search Strategy

The studies containing information on long-COVID or post-COVID were identified via PubMed from 1 February 2020 - 26 October 2022 using the keywords "((long?covid) OR (post?covid)) AND (obsver* OR cohort* OR cross?section* OR case?control OR longitud* OR case?report OR case?series)".

The Preferred Reporting Items for Systematic Reviews and Meta-Analyses (PRISMA) 2020 guidelines were implemented to describe the search strategy of this study

.

© 2023 Chaichana U et al. *JAMA Network Open.*
