# Supplementary material for: Definition of Post–COVID-19 Condition Among Published Research Studies
Source: JAMA Netw Open. 2023 Apr 5;6(4):e235856. doi: 10.1001/jamanetworkopen.2023.5856 (PMC10077105; doi:10.1001/jamanetworkopen.2023.5856)
Supplement: Supplement 2. — Data Sharing Statement [file jamanetwopen-e235856-s002.pdf]

Data Sharing Statement

Chaichana. Definition of Post-COVID-19 Condition Among Published Research Studies. *JAMA Netw Open*. Published April 05, 2023. doi:10.1001/jamanetworkopen.2023.5856

Data
Data available: No
